# Combined impact of the inter and intra-patient variability of tacrolimus blood level on allograft outcomes in kidney transplantation

**DOI:** 10.3389/fimmu.2022.1037566

**Published:** 2022-11-16

**Authors:** Yohan Park, Hanbi Lee, Sang Hun Eum, Eun Jeong Ko, Ji Won Min, Se-Hee Yoon, Won-Min Hwang, Sung-Ro Yun, Chul Woo Yang, Jieun Shin, Byung Ha Chung

**Affiliations:** ^1^ Division of Nephrology, Department of Internal Medicine, Konyang University Hospital, College of Medicine, Konyang University, Daejeon, South Korea; ^2^ Transplantation Research Center, Seoul St. Mary’s Hospital, College of Medicine, The Catholic University of Korea, Seoul, South Korea; ^3^ Division of Nephrology, Department of Internal Medicine, Seoul St. Mary’s Hospital, College of Medicine, The Catholic University of Korea, Seoul, South Korea; ^4^ Division of Nephrology, Department of Internal Medicine, Incheon St. Mary’s Hospital, College of Medicine, The Catholic University of Korea, Seoul, South Korea; ^5^ Division of Nephrology, Department of Internal Medicine, Bucheon St. Mary’s Hospital, College of Medicine, The Catholic University of Korea, Seoul, South Korea; ^6^ Department of Biomedical Informatics, College of Medicine, Konyang University, Nonsan, South Korea

**Keywords:** allograft, metabolism, transplant, graft survival, tacrolimus

## Abstract

**Introduction:**

Tacrolimus (TAC) has been widely used as an immunosuppressant after kidney transplantation (KT); however, the combined effects of intra-patient variability (IPV) and inter-patient variability of TAC-trough level (C0) in blood remain controversial. This study aimed to determine the combined impact of TAC-IPV and TAC inter-patient variability on allograft outcomes of KT.

**Methods:**

In total, 1,080 immunologically low-risk patients who were not sensitized to donor human leukocyte antigen (HLA) were enrolled. TAC-IPV was calculated using the time-weighted coefficient variation (TWCV) of TAC-C0, and values > 30% were classified as high IPV. Concentration-to-dose ratio (CDR) was used for calculating TAC inter-patient variability, and CDR < 1.05 ng•mg/mL was classified as rapid metabolizers (RM). TWCV was calculated based on TAC-C0 up to 1 year after KT, and CDR was calculated based on TAC-C0 up to 3 months after KT. Patients were classified into four groups according to TWCV and CDR: low IPV/non-rapid metabolizer (NRM), high IPV/NRM, low IPV/RM, and high IPV/RM. Subgroup analysis was performed for pre-transplant panel reactive antibody (PRA)-positive and -negative patients (presence or absence of non-donor-specific HLA-antibodies). Allograft outcomes, including deathcensored graft loss (DCGL) and biopsy-proven allograft rejection (BPAR), were compared.

**Results:**

The incidences of DCGL, BPAR, and overall graft loss were the highest in the high-IPV/RM group. In addition, a high IPV/RM was identified as an independent risk factor for DCGL. The hazard ratio of high IPV/RM for DCGL and the incidence of active antibody-mediated rejection were considerably increased in the PRA-positive subgroup.

**Discussion:**

High IPV combined with RM (inter-patient variability) was closely related to adverse allograft outcomes, and hence, more attention must be given to pre-transplant PRA-positive patients.

## Introduction

Tacrolimus (TAC) is the most widely used maintenance immunosuppressant for kidney transplantation (KT) and is recommended as a first-line calcineurin inhibitor (CNI) (1–3). However, as TAC has a very narrow therapeutic range, it is important to monitor its trough levels (C0) in patients to maintain an appropriate drug dose. Kidney Disease: Improving Global Outcomes guideline identifies only target TAC-C0 after KT; however, even if the mean TAC-C0 is within the target range, there is a risk of high or low drug exposure if the fluctuation in each TAC-C0 is large (3).

The intra-patient variability (IPV) of TAC has been used as an index that can reflect TAC-C0 fluctuations. Many recent studies, including ours, have reported that a high IPV of TAC-C0 is associated with rejection, *de novo* donor-specific antibody (DSA) development, and adverse long-term allograft outcomes (4–8).

TAC metabolism varies from patient to patient, thereby affecting TAC blood concentrations, which is referred to as inter-patient variability. The cytochrome P450 (CYP) 3A5 isoenzyme plays a major role in TAC metabolism, showing differences in metabolic characteristics according to genotype (9, 10). However, it is difficult to regularly test for the CYP3A5 genotype in all patients in actual clinical practice. Therefore, the concentration-to-dose ratio (CDR) of TAC has been proposed as an easy indicator of TAC metabolism in clinical practice (11). Previous studies have reported that CDRs correlate with genetic variants of CYP3A5 genotypes and that patients with CDR <1.05 ng•mg/mL can be classified as rapid metabolizers (RMs) (12, 13).

In RM, a higher drug dose is required to maintain the same TAC-C0, which increases the peak blood TAC level while lowering TAC-C0, which may cause adverse allograft outcomes (14). Several recent studies on CDR have reported that RM is a significant factor affecting the prognosis of KT recipients (11, 13). However, some studies have also reported that CDR is not a significant factor influencing allograft outcomes; therefore, the importance of CDR in KT remains controversial (15, 16). In patients showing high IPV in RM status, fluctuations of administered drug doses increase, which may cause not only increasing fluctuation of TAC-C0 but also TAC peak concentrations, which may have a greater impact on adverse allograft outcomes. However, this combined effect of inter-patient variability and IPV has been reported in a small group study to date; therefore, it has not yet been fully investigated (17).

Previously, we reported the adverse impact of high IPV on allograft outcomes, including the development of allograft rejection and failure in a large-scale, long-term follow-up study (8). In the present study, we aimed to analyze the combined effect of CDR and TAC IPV on long-term allograft outcomes in the same patient cohort.

## Materials and methods

### Study design

A single-center, retrospective, and observational cohort study was conducted, and data were collected using a clinical data warehouse system. A total of 1,227 patients underwent KT at Seoul St. Mary’s Hospital between January 2010 and December 2018. Out of these, patients who experienced allograft loss within 90 days (n = 7), who were sensitized to donor human leukocyte antigen (HLA) before KT (n = 126), and whose CDR calculation was not possible due to missing TAC-C0 results (n = 14) were excluded from the present study. Sensitization to donor HLA was defined as positivity to the complement-dependent cytotoxicity cross-match test, flow cytometry cross-match test, or the presence of DSA with a median fluorescence intensity (MFI) of ≥ 1,000 before KT (18). Ultimately, 1,080 patients were included in the study, the last follow-up date was December 31, 2020, and the mean follow-up duration of these patients was 5.7 years.

**Figure 1:**
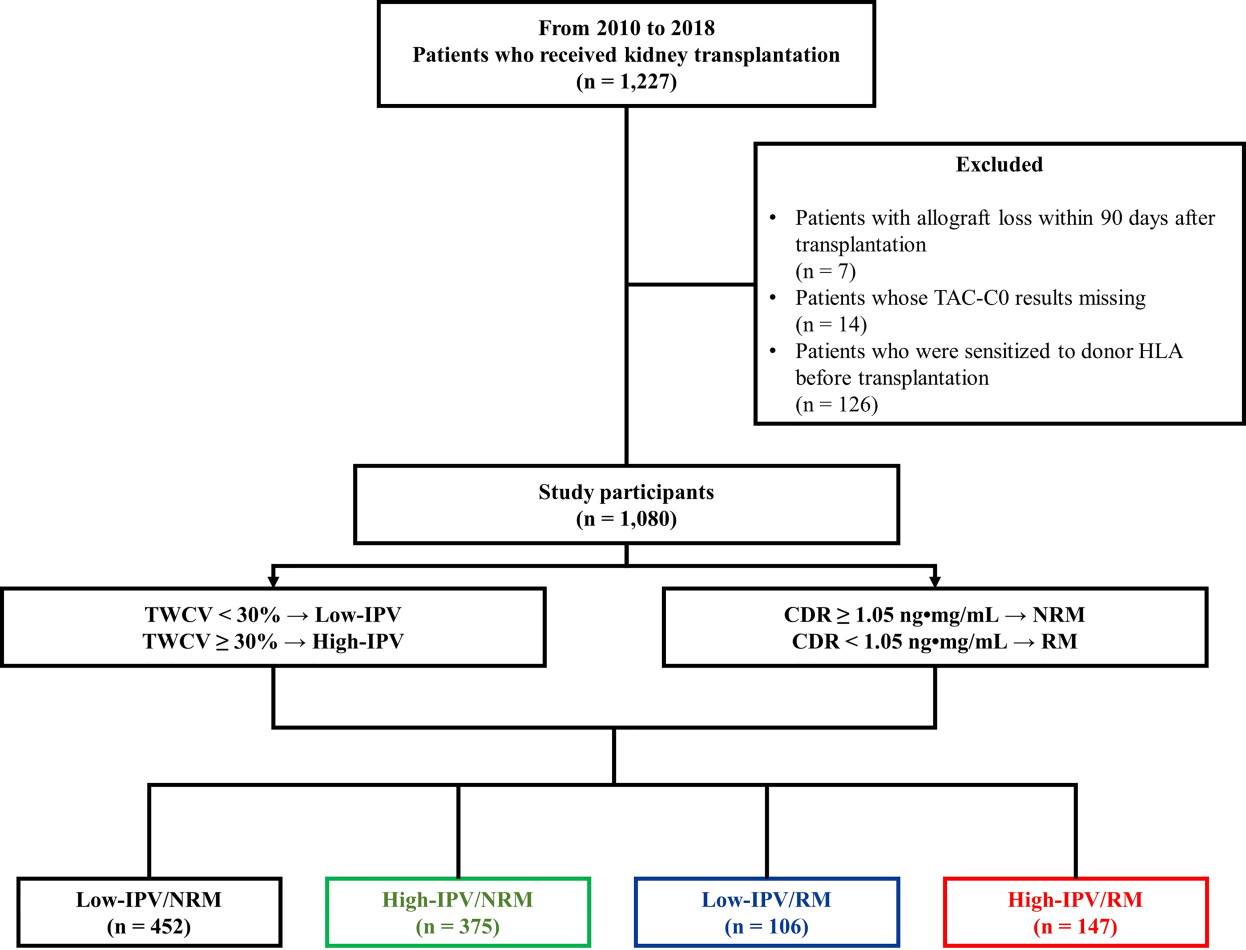
Study design. Between January 2010 and December 2018, 1,080 patients were included in the study. Based on a TWCV of 30%, the patients were classified into high- or low-IPV groups. The RM and NRM groups were classified based on the CDR 1.05 ng•mg/mL. Finally, the patients were classified into four groups according to their TAC-IPV and RM/NRM status. CDR, concentration-to-dose ratio; HLA, human leukocyte antigen; IPV, intra-patient variability; NRM, non-rapid metabolizer; RM, rapid metabolizer; TAC-C0, tacrolimus trough level; TWCV, time-weighted coefficient variation.

TAC-IPV was calculated using time-weighted coefficient of variability (TWCV). Previous studies have reported that a coefficient of variation > 30% results in adverse allograft outcomes, and hence, patients were classified into high- or low-IPV groups based on a TWCV ≥ 30% or < 30%, respectively (4, 7, 8). In previous studies, a CDR of 1.05 ng•mg/mL was considered a cut-off value between RMs and non-rapid metabolizers (NRMs) (12, 13); patients were classified as either RM (CDR < 1.05 ng•mg/mL) or NRM (CDR ≥ 1.05 ng•mg/mL). Thereafter, the patients were classified into four groups: low-IPV/NRM, high-IPV/NRM, low-IPV/RM, and high-IPV/RM groups (**Figure 1**). In addition, we performed a subgroup analysis according to panel-reactive antibody (PRA) positivity at baseline. Within PRA-positive and PRA-negative subgroups, we divided the patients into four groups according to IPV and CDR. This study was performed in accordance with the principles of the Declaration of Helsinki and approved by the institutional review board of Seoul St. Mary’s Hospital (XC20WIDI0024K).

### TAC-C0-TWCV and CDR calculation

**Figure 2:**
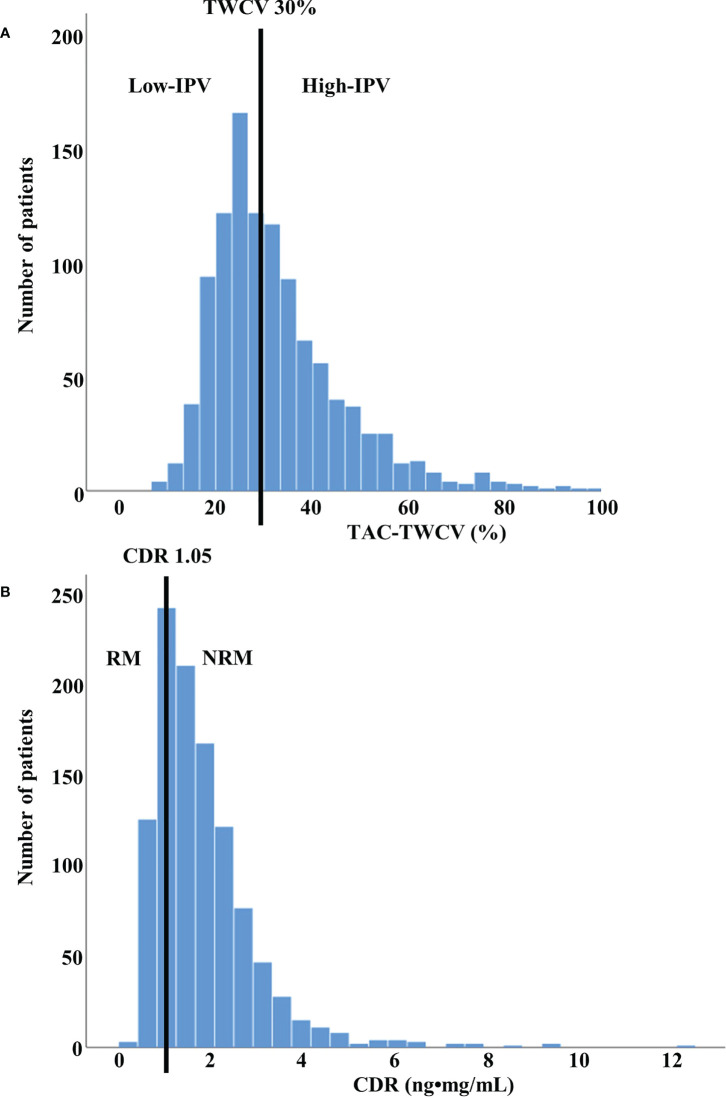
Distribution of TWCV and CDR values in study population **(A)** The distribution of TWCV in the study population for the first year after transplant using TAC-C0. The patients were classified into low- and high-IPV groups based on TWCV values of ≥30% or <30%, respectively. The low-IPV and high-IPV groups included 558 and 522 patients, respectively. **(B)** The distribution of CDR in the study population for the first 3 months after transplant using TAC-C0. Patients with CDR > 1.05 ng•mg/mL and CDR < 1.05 ng•mg/mL were classified as NRM and RM, respectively. NRM and RM groups included 827 and 253 patients, respectively. CDR, concentration-to-dose ratio; IPV, intra-patient variability; NRM, non-rapid metabolizer; RM, rapid metabolizer; TAC-C0, tacrolimus trough level; TWCV, time-weighted coefficient variation.

Blood TAC levels were measured using the automated dimension TAC method (Siemens Healthcare Diagnostics Inc., Deerfield, IL, USA), which is an affinity chrome-mediated immunoassay (19). The test was performed in a fasting state just before administering the next TAC dose at the outpatient clinic. Therefore, TAC-C0s during hospitalization after KT were not used in the analysis. Rather, TAC-C0s from a median of 22 days (interquartile range: 20–26 days) after KT—which is the start of the outpatient clinic visit—were analyzed. To correct for the difference in time-interval of outpatient clinic visits according to the period after KT, TAC-C0-TWCV was calculated as previously reported (5, 8). Briefly, the time-weighted average (TWA) of TAC-C0 was calculated using the following formula: $TW\mu =\frac{1}{t}\sum_{n=1}^{i}x_{i}t_{i}$ . The time-weighted standard deviation was calculated using the following formula: $TW\sigma =\sqrt{\frac{1}{t}\sum_{n=1}^{i}{\left(x_{i}-µ\right)}^{2}t_{i}}$ where *i* is the patient’s visit to the *i*-th outpatient clinic after transplantation *x_i_* is TAC-C0 (ng/mL) during the interval period, *t*_*i*_ is the time interval (days), and *t* is the total duration of drug exposure (days). TAC-C0-TWCV was calculated as a percentage using the formula $\frac{TW\sigma }{TW\mu }\times 100$ . TWCV was calculated using TAC-C0 up to 1 year after KT. The CDR was calculated by dividing TAC-C0 with the previously administered TAC dose at every outpatient visit, and the average value of CDRs for 3 months after KT was used. Since CDR values can be calculated from the second outpatient visit, the specific period for CDR values ranged from a median of 29 days (interquartile range, 27–33 days) to 3 months after KT. The distributions of the TWCV and CDR in the patients included in this study are shown in **Figure 2**.

### Immunosuppressive regimen

The maintenance immunosuppressive therapy includes TAC, mycophenolate mofetil (MMF), and glucocorticoids (prednisolone or deflazacort). The initial TAC dosage was 0.1 mg/kg, which was divided into twice-daily doses of 0.05 mg/kg each and administered from 2 days before KT. The target TAC-C0 was 8–12 ng/mL until 3 months after KT and 5–8 ng/mL thereafter. The initial MMF dose was 1,500 mg, which was also divided into twice daily doses of 750 mg each and administered from 2 days before KT. In case of enteric-coated mycophenolate sodium formulation, 1,080 mg was administered as twice daily doses of 540 mg each. Intravenous (IV) glucocorticoids were administered at a high dose during the perioperative period (IV methylprednisolone; 1000 mg on the operation day, tapering to 60 mg on post-operation day 4). The dose was then reduced (5 mg prednisolone or 6 mg deflazacort once daily within 3 months after KT). Based on the patient’s immunological risk (re-transplant or positivity to PRAs), either 1.5 mg/kg of IV rabbit anti-thymocyte globulin was administered for five consecutive days from day 0 to 4 or 20 mg of anti-interleukin-2 receptor antagonist (basiliximab) was administered on days 0 and 4. Patients with ABO-incompatible KT received desensitization therapy as previously reported (20).

### Clinical parameters

All data were obtained from a clinical data warehouse system. Information about baseline characteristics, including the age, sex, body mass index of the donor and the recipient as well as the causes of end-stage kidney disease (ESKD), dialysis-related factors, and transplant-related factors of the recipient were collected. In addition, allograft outcome parameters, including data regarding the development of *de novo* DSA, biopsy-proven allograft rejection (BPAR), CNI toxicity, and death-censored allograft loss (DCGL) as well as mortality rates were collected.

### Clinical outcomes

The primary outcome of this study was DCGL, and the secondary outcomes were the development of *de novo* DSA, BPAR, CNI toxicity, and mortality rates. DCGL included the cases when dialysis or re-transplantation was required due to allograft failure, excluding patient death with a functioning allograft. Mortality included due to any cause following KT. We compared the primary and secondary outcomes among the four groups for the entire cohort as well as for the subgroups based on PRA positivity.

Allograft kidney biopsy was performed in cases of unexpected allograft dysfunction (when the serum creatinine level was 20% above the baseline), unexpected development of proteinuria, and occurrence of *de novo* DSA. Allograft renal biopsy findings were interpreted according to the 2019 Banff classification. Biopsy-proven rejection was diagnosed *via* allograft biopsy for acute T-cell mediated rejection (TCMR), active antibody-mediated rejection (ABMR), chronic active TCMR, and chronic active ABMR. CNI toxicity and BK virus nephropathy were diagnosed based on the Banff classification (21–23). Donor-specific anti-HLA antibodies (HLA-DSAs) were detected using Lifecodes LSA Class I and II kits or the LABScreen Single Antigen kit as previously described (24). A positive result was defined as an MFI ≥ 1,000. HLA-DSA monitoring (3 and 12 months posttransplant and annually thereafter) was performed. Moreover, HLA-DSA detection was performed according to the judgment of the clinician in case of unexpected allograft dysfunction or occurrence of proteinuria.

### Statistical analysis

Continuous variables were expressed as mean ± standard deviation. As the continuous variables did not follow normal distribution, the Kruskal–Wallis test was performed. Wilcoxon’s rank-sum test, followed by the Bonferroni method, was performed for *post hoc* analysis. All categorical variables were compared using the chi-square test or Fisher’s exact test and expressed as proportions. Death-censored graft survival and patient survival were analyzed using Kaplan–Meier curves, and a between-group comparison was performed using the log-rank test. The effects of TAC-IPV with RM or NRM status on DCGL were analyzed using the Cox proportional hazards regression model. We developed a multivariate model using all the significant baseline characteristics (TAC-C0-TWA, age, sex, BMI, and induction regimen) among the groups. In addition, subgroup analysis was performed according to pre-transplant immunological risk, which was based on PRA positivity or negativity. All unavailable data of a patient were censored, and only data up till the last follow-up date were retained. Statistical significance was set at P < 0.05. All statistical analyses were performed using the SAS^®^ version 9.4 software (SAS Institute, Inc., Cary, NC, USA).

## Results

### Comparison of baseline characteristics according to TAC-IPV and RM/NRM status

Baseline characteristics of the donors and recipients were compared among the four groups based on TAC IPV and RM/NRM status in the overall cohort, as well as PRA-positive, and -negative subgroups (**Table 1** and **Supplemental Table 1**, and **Supplemental Table 2**). TAC measurement frequency was significantly higher in the high IPV groups. TWA of TAC-C0 was significantly lower in the RM groups compared to NRM groups, and it was the lowest for the high-IPV/RM group (5.24 ± 1.42 ng/mL). Furthermore, the age of patients was lower in the high-IPV/RM group and the proportion of males was lower in the high-IPV/NRM group. The proportion of patients using basiliximab as an induction regimen was lower in the low-IPV/NRM group. The proportion of PRA positivity was 34.7% (n = 157) in the low-IPV/NRM group, 30.7% (n = 115) in the high-IPV/NRM group, 27.4% (n = 29) in the low-IPV/RM group, and 32.0% (n = 47) in the high-IPV/RM group, which was not significantly different. Other baseline characteristics, including the proportion of ABO-incompatible KT, also showed no significant differences among the four groups.

**Table 1:** Baseline characteristics according to TAC-IPV and RM/NRM status.

	Low-IPV/NRM(n = 452)	High-IPV/NRM(n = 375)	Low-IPV/RM(n = 106)	High-IPV/RM(n = 147)	P-value
**Donor factors**					
Age (years)	44.7 ± 12.4	45.0 ± 12.9	44.3 ± 12.5	44.6 ± 13.8	0.951
Male sex (n, %)	225 (49.8%)	190 (50.7%)	57 (53.8%)	79 (53.7%)	0.788
BMI (kg/m^2^)	23.6 ± 3.4	23.7 ± 3.4	23.7 ± 3.7	23.8 ± 3.7	0.929
**Transplant information**					
Preemptive KT (n, %)	98 (21.7%)	82 (21.9%)	19 (17.9%)	35 (23.8%)	0.734
Deceased donor KT (n, %)	154 (34.1%)	140 (37.3%)	37 (34.9%)	42 (28.6%)	0.301
ABO-incompatible KT (n, %)	69 (15.3%)	56 (14.9%)	13 (12.3%)	26 (17.7%)	0.384
Previous history of KT (n, %)	11 (2.4%)	5 (1.3%)	1 (0.9%)	6 (4.1%)	0.192
PRA positive (n, %)	157 (34.7%)	115 (30.7%)	29 (27.4%)	47 (32.0%)	0.411
Cold ischemic time (min)	188 ± 94	188 ± 74	204 ± 89	213 ± 84	0.318
Mismatch number	3.45 ± 1.55	3.43 ± 1.58	3.29 ± 1.62	3.48 ± 1.50	0.834
**Tacrolimus-related information**					
Tacrolimus measurement frequency	15.1 ± 2.9^†§^	15.7 ± 3.1^*‡^	14.9 ± 2.4^†§^	16.0 ± 3.7^*‡^	<0.001
TAC-C0-TWA (ng/mL)	6.94 ± 1.22^†‡§^	6.50 ± 1.71^*‡§^	5.94 ± 1.59^*†§^	5.24 ± 1.42^*†‡^	<0.001
TWCV (%)	22.7 ± 4.5^†§^	43.6 ± 13.3^*‡^	23.4 ± 4.5^†§^	41.6 ± 10.9^*‡^	<0.001
CDR (ng•mg/mL)	2.16 ± 1.15^‡§^	2.13 ± 1.12^‡§^	0.82 ± 0.16^*†^	0.80 ± 0.17^*†^	<0.001
**Recipient factors**					
Age (years)	48.6 ± 11.1^§^	47.0 ± 11.8^§^	45.7 ± 10.9	42.5 ± 11.6^*†^	<0.001
Male sex (n, %)	302 (66.8%)^†^	206 (54.9%)^*‡§^	76 (71.7%)^†^	89 (60.5%)^†^	0.001
BMI (kg/m^2^)	23.2 ± 3.6	22.9 ± 3.5	23.8 ± 3.7	22.8 ± 3.6	0.032
Cause of ESKD					
DM (n, %)	96 (21.2%)	76 (20.3%)	26 (24.5%)	24 (16.3%)	0.426
HTN (n, %)	61 (13.5%)	55 (14.7%)	19 (17.9%)	19 (12.9%)	0.649
CGN (n, %)	154 (34.1%)	133 (35.5%)	36 (34.0%)	52 (35.4%)	0.972
Others (n, %)	59 (13.1%)	41 (10.9%)	9 (8.5%)	18 (12.2%)	0.552
Unknown (n, %)	82 (18.1%)	70 (18.7%)	16 (15.1%)	34 (23.1%)	0.408
Dialysis information					
Hemodialysis (n, %)	275 (60.8%)	233 (62.1%)	68 (64.2%)	80 (54.4%)	0.350
Peritoneal dialysis (n, %)	79 (17.5%)	60 (16.0%)	19 (17.9%)	32 (21.8%)	0.487
Dialysis vintage (months)	46.0 ± 55.8	43.7 ± 49.4	37.1 ± 49.5	37.0 ± 52.1	0.143
**Induction regimen**					
Antithymocyte globulin (n, %)	91 (20.1%)	60 (16.0%)	16 (15.1%)	16 (10.9%)	0.055
Basiliximab (n, %)	361 (79.9%)^§^	318 (84.8%)	92 (86.8%)	134 (91.2%)^*^	0.007

Continuous variables are shown as mean ± standard deviation and categorical variables are shown as proportions.^†^P <0.0083 versus high-IPV/NRM group^§^P <0.0083 versus high-IPV/RM group.

BMI, body mass index; CDR, concentration-to-dose ratio; CGN, clinical glomerulonephritis; DM, diabetes mellitus; ESKD, end-stage kidney disease; HTN, hypertension; IPV, intra-patient variability; KT, kidney transplantation; NRM, non-rapid metabolizer; PRA, panel reactive antibody; RM, rapid metabolizer; TAC, tacrolimus; TAC-C0, tacrolimus trough level; TWA, time-weighted average; TWCV, time-weighted coefficient variation.

### Comparison of the incidence of BPAR and other outcomes according to TAC-IPV and RM/NRM status

**Table 2** shows the comparison of complications, including BPAR, for the four groups that were divided based on TAC-IPV and RM/NRM status. The overall incidence of BPAR was the highest in the high-IPV/RM group, followed by the high-IPV/NRM group. In the rejection subtype analysis, the incidences of acute TCMR and active ABMR were higher in the high-IPV/RM group compared to those in the low-IPV/NRM group. The incidence of chronic active TCMR did not differ among the four groups, but the incidence of chronic active ABMR was higher in the high-IPV groups, regardless of RM/NRM status. CNI toxicity tended to be higher in the high-IPV/RM group, but this observation was not statistically significant. The incidence of BK nephropathy and *de novo* DSA development did not differ among the four groups.

**Table 2:** Incidences of BPAR and other outcomes according to TAC-IPV and RM/NRM status.

	Low-IPV/NRM(n = 452)	High-IPV/NRM(n = 375)	Low-IPV/RM(n = 106)	High-IPV/RM(n = 147)	P-value
**Overall BPAR** (n, %)	64 (14.2%)^†§^	82 (21.9%)^*^	19 (17.9%)	47 (32.0%)^*^	<0.001
Acute TCMR (n, %)	53 (11.7%)^§^	66 (17.6%)	16 (15.1%)	37 (25.2%)^*^	0.001
Active ABMR (n, %)	13 (2.9%)^§^	16 (4.3%)	40 (3.8%)	15 (10.2%)^*^	0.003
Chronic active TCMR (n, %)	6 (1.3%)	3 (0.8%)	0 (0.0%)	1 (0.7%)	0.584
Chronic active ABMR (n, %)	5 (1.1%)^†§^	16 (4.3%)^*^	0 (0.0%)	7 (4.8%)^*^	0.003
***De novo* DSA positive** (n, %)	49 (10.8%)	46 (12.3%)	12 (11.3%)	23 (15.7%)	0.477
**CNI toxicity** (n, %)	68 (15.0%)	58 (15.5%)	15 (14.2%)	34 (23.1%)	0.106
**BK virus nephropathy** (n, %)	11 (2.4%)	18 (4.8%)	4 (3.8%)	5 (3.4%)	0.333

Categorical variables are shown as proportions. ^*^P <0.0083 versus low-IPV/NRM group, ^†^P <0.0083 versus high-IPV/NRM group, ^§^P <0.0083 versus high-IPV/RM group.

ABMR, antibody-mediated rejection; BPAR, biopsy-proven allograft rejection; CNI, calcineurin inhibitor; DSA, donor-specific antibody; IPV, intra-patient variability; NRM, non-rapid metabolizer; RM, rapid metabolizer; TAC, tacrolimus; TCMR, T-cell mediated rejection.

### Comparison of the incidence of DCGL and mortality rates according to TAC-IPV and RM/NRM status

**Figure 3:**
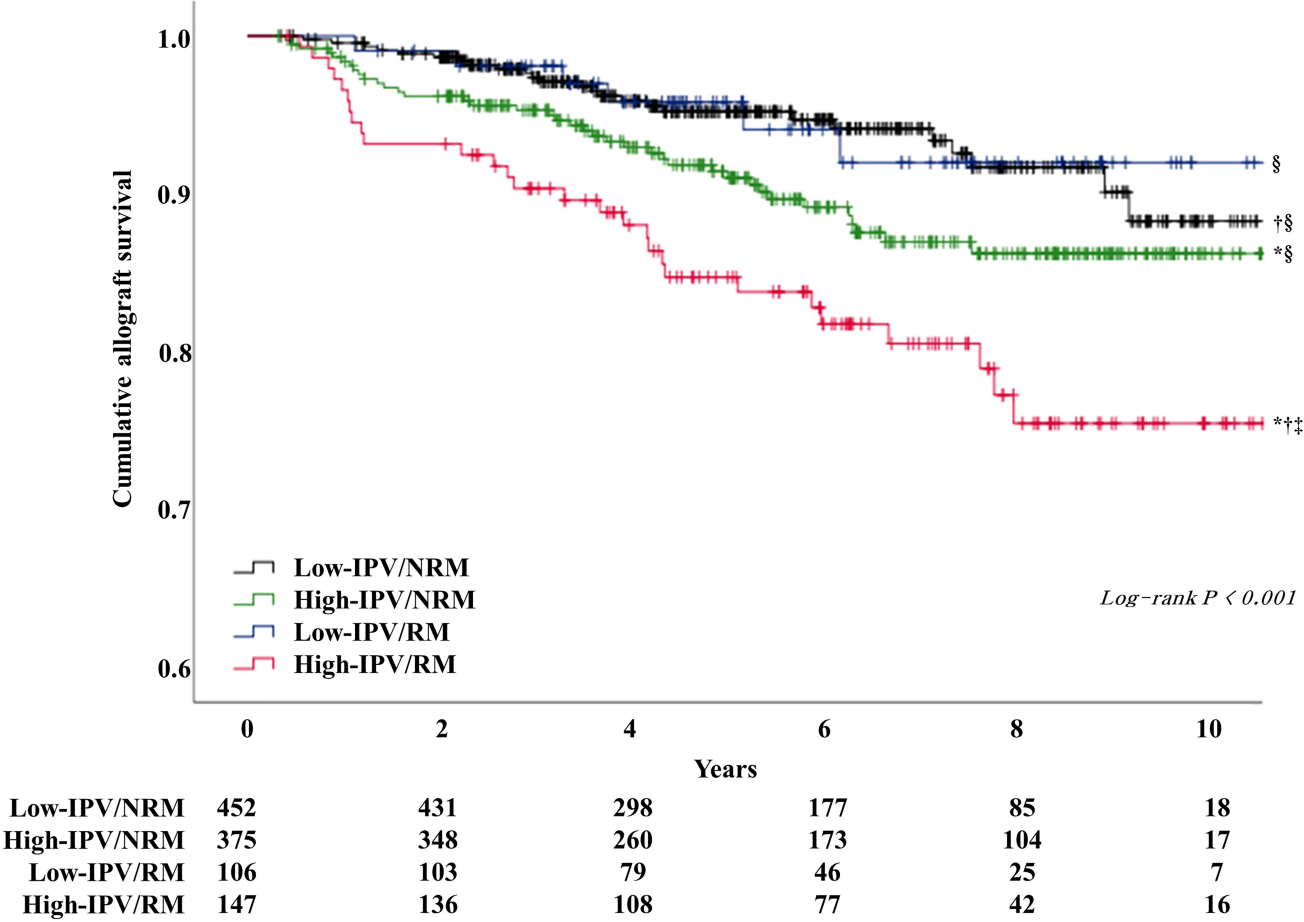
Kaplan–Meier survival curve analysis of allograft survival according to TAC-IPV and RM/NRM status. The high IPV/RM group had the lowest allograft survival rate. The high-IPV/NRM group showed intermediate outcomes, and the low-IPV group showed relatively good allograft survival rates regardless of NRM/RM status (log-rank P < 0.001). ^*^Log-rank P <0.05 versus low-IPV/NRM group; ^†^log-rank P <0.05 versus high-IPV/NRM group; ^‡^log-rank P <0.05 versus low-IPV/RM group; ^§^log-rank P <0.05 versus high-IPV/RM group.

The incidence of DCGL was 5.5% (n = 25), 10.1% (n = 38), 5.7% (n = 6), and 19.0% (n = 28) in the low-IPV/NRM, high-IPV/NRM, low-IPV/RM, and high-IPV/RM groups, respectively (P < 0.001). The incidence of DCGL was significantly higher in the high IPV/RM group, compared to that in the other groups. The mortality rates were 5.3% (n = 24) in the low-IPV/NRM group, 4.5% (n = 17) in the high-IPV/NRM group, 7.5% in the low-IPV/RM group (n = 8), and 6.1% (n = 9) in the high-IPV/RM group, with no significant difference among these groups (**Table 3**). The Kaplan–Meier survival curve showed that the allograft survival was lowest in the high-IPV/RM group, followed by the high-IPV/NRM group. No significant differences were observed in the low-IPV groups, regardless of the RM/NRM status (**Figure 3**).

**Table 3:** DCGL, overall graft loss, and mortality rates according to TAC-IPV and RM/NRM status.

	Low-IPV/NRM (n = 452)	High-IPV/NRM(n = 375)	Low-IPV/RM(n = 106)	High-IPV/RM(n = 147)	P-value
**DCGL** (n, %)	25 (5.5%)^§^	38 (10.1%)^§^	6 (5.7%)^§^	28 (19.0%)^*†‡^	<0.001
**Overall graft loss** (n, %)	43 (9.5%)^§^	51 (13.6%)	13 (12.3%)	32 (21.8%)^*^	0.002
**Mortality** (n, %)	24 (5.3%)	17 (4.5%)	8 (7.5%)	9 (6.1%)	0.643

Categorical variables are shown as proportions. ^*^P <0.0083 versus low-IPV/NRM group, ^†^P <0.0083 versus high-IPV/NRM group, ^‡^P <0.0083 versus low-IPV/RM group, ^§^P <0.0083 versus high-IPV/RM group.

DCGL, death-censored graft loss; IPV, intra-patient variability; NRM, non-rapid metabolizer; RM, rapid metabolizer; TAC, tacrolimus.

### Comparison of allograft outcomes according to TAC-IPV and RM/NRM status in PRA-positive and -negative subgroups

**Figure 4:**
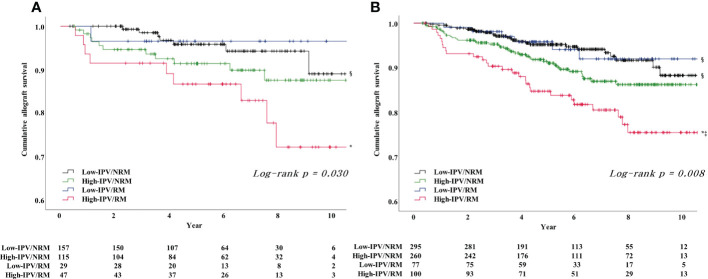
Result of Kaplan–Meier survival analysis for allograft survival for each PRA-positive and -negative subgroups based on TAC-IPV and RM/NRM status. **(A)** Allograft survival in the PRA-positive subgroup was the lowest in the high-IPV/RM group, and the allograft survival rate was remarkably reduced compared to the rates of the overall cohort and PRA-negative subgroup. **(B)** Allograft survival in the PRA negative subgroup was the lowest in the high-IPV/RM group, and the allograft survival rate was comparable with those of the overall cohort. ^*^Log-rank P <0.05 versus low-IPV/NRM group; ^‡^log-rank P <0.05 versus low-IPV/RM group; ^§^log-rank P <0.05 versus high-IPV/RM group.

The subgroup analysis, which was performed according to PRA positivity or negativity, showed that the incidence of DCGL was significantly higher in the high-IPV/RM group, compared to that in the low-IPV/NRM group (**Table 4**). Furthermore, the Kaplan–Meier survival analysis showed that allograft survival was the lowest in the high-IPV/RM group irrespective of PRA positivity; this observation was similar for the overall cohort (**Figures 4A, B**). In the PRA-negative group, the incidence of active ABMR did not differ among the four groups; however, in the PRA-positive subgroup, the incidence of active ABMR was significantly higher in the high-IPV/RM group than in the other groups (19.2% in the high-IPV/RM group vs. 5.5%, 3.2%, and 4.4% in the other groups, **Table 4**).

**Table 4:** Incidences of BPAR and other outcomes according to TAC-IPV and RM/NRM status in each PRA positive and negative subgroups.

	Low-IPV/NRM	High-IPV/NRM	Low-IPV/RM	High-IPV/RM	P-value
**PRA positive subgroup**	**n = 157**	**n = 115**	**n = 29**	**n = 47**	
**DCGL** (n, %)	7 (4.5%)^§^	11 (9.6%)	1 (3.4%)	9 (19.1%)^*^	0.008
**Overall BPAR** (n, %)	14 (8.9%)^§^	22 (19.1%)	4 (13.8%)	14 (29.8%)^*^	0.003
Acute TCMR (n, %)	10 (6.4%)	17 (14.8%)	4 (13.8%)	8 (17.0%)	0.073
Active ABMR (n, %)	5 (3.2%)^§^	5 (4.4%)^§^	0 (0.0%)	9 (19.2%)^*†^	<0.001
Chronic active TCMR (n, %)	0 (0.0%)	1 (0.9%)	0 (0.0%)	1 (2.1%)	0.357
Chronic active ABMR (n, %)	1 (0.6%)	3 (2.6%)	0 (0.0%)	3 (6.4%)	0.076
***De novo* DSA positive** (n, %)	20 (12.7%)	14 (12.2%)	1 (3.5%)	10 (21.3%)	0.152
**CNI toxicity** (n, %)	23 (14.7%)	20 (17.4%)	2 (6.9%)	10 (21.3%)	0.367
**BK virus nephropathy** (n, %)	3 (1.9%)	7 (6.1%)	2 (6.9%)	2 (4.3%)	0.295
**PRA negative subgroup**	**n = 295**	**n = 260**	**n = 77**	**n = 100**	
**DCGL** (n, %)	18 (6.1%)^§^	27 (10.4%)	5 (6.5%)	19 (19.0%)^*^	0.001
**Overall BPAR** (n, %)	50 (17.0%)^§^	60 (23.1%)	15 (19.5%)	33 (33.0%)^*^	0.007
Acute TCMR (n, %)	43 (14.6%)^§^	49 (18.9%)	12 (15.6%)	29 (29.0%)^*^	0.012
Active ABMR (n, %)	8 (2.7%)	11 (4.2%)	4 (5.2%)	6 (6.0%)	0.447
Chronic active TCMR (n, %)	6 (2.0%)	2 (0.8%)	0 (0.0%)	0 (0.0%)	0.202
Chronic active ABMR (n, %)	4 (1.4%)^†§^	13 (5.0%)^*^	0 (0.0%)	4 (4.0%)^*^	0.025
***De novo* DSA positive** (n, %)	29 (9.8%)	32 (12.3%)	11 (14.3%)	13 (13.0%)	0.624
**CNI toxicity** (n, %)	45 (15.3%)	38 (14.6%)	13 (16.9%)	24 (24.0%)	0.164
**BK virus nephropathy** (n, %)	8 (2.7%)	11 (4.2%)	2 (2.6%)	3 (3.0%)	0.758

Categorical variables are shown as proportions. ^*^P <0.0083 versus low-IPV/NRM group, ^†^P <0.0083 versus high-IPV/NRM group.

ABMR, antibody-mediated rejection; BPAR, biopsy-proven allograft rejection; CNI, calcineurin inhibitor; DCGL, death-censored graft loss; DSA, donor-specific antibody; IPV, intra-patient variability; NRM, non-rapid metabolizer; PRA, panel reactive antibody; RM, rapid metabolizer; TAC, tacrolimus; TCMR, T-cell mediated rejection.

### Multivariate Cox proportional hazards regression model analysis for DCGL

**Table 5** shows the results of the multivariate Cox regression analysis of DCGL according to TAC-IPV and NRM/RM status. TAC-C0-TWA, which is a well-known factor influencing allograft outcome, was included as the main adjustment factor in the regression models. In the overall cohort analysis, high-IPV/RM was an independent risk factor in all regression models after adjusting for factors that showed differences in baseline characteristics including TAC-C0-TWA (age, sex, BMI, and induction regimen) (hazard ratio [HR] 3.06 [1.78–5.25] in model 1, HR 2.50 [1.38–4.52] in model 2, HR 2.47 [1.34–4.56] in model 3). In the PRA-positive and -negative subgroup analysis, multivariate Cox regression analysis showed that a high IPV/RM was an independent risk factor for DCGL in all regression models, similar to the results in the overall cohort. The HR of high-IPV/RM in the PRA-positive subgroup was higher than that in the overall cohort and PRA-negative subgroup (HR 3.80 in the PRA-positive subgroup vs. HR 2.47 in the overall cohort and HR 2.12 in the PRA-negative subgroup).

**Table 5:** Multivariate Cox proportional hazard regression model analysis for DCGL.

		Hazard ratio (95% confidence interval)			
		Low-IPV/NRM	High-IPV/NRM	Low-IPV/RM	High-IPV/RM
**Overall cohort**	Model 1	Ref.	1.73 (1.04–2.86)	0.94 (0.39–2.29)	3.06 (1.78–5.25)
	Model 2	Ref.	1.60 (0.95–2.67)	0.83 (0.34–2.06)	2.50 (1.38–4.52)
	Model 3^*^	Ref.	1.64 (0.98–2.77)	0.82 (0.33–2.03)	2.47 (1.34–4.56)
**PRA positive subgroup**	Model 1	Ref.	1.98 (0.77–5.12)	0.71 (0.09–5.79)	3.76 (1.40–10.13)
	Model 2	Ref.	1.91 (0.72–5.07)	0.68 (0.08–5.63)	3.54 (1.22–10.24)
	Model 3^†^	Ref.	1.96 (0.73–5.22)	0.68 (0.08–5.65)	3.80 (1.27–11.37)
**PRA negative subgroup**	Model 1	Ref.	1.62 (0.89–2.94)	0.97 (0.36–2.61)	2.79 (1.47–5.33)
	Model 2	Ref.	1.48 (0.80–2.71)	0.83 (0.30–2.28)	2.10 (1.02–4.33)
	Model 3^‡^	Ref.	1.43 (0.77–2.64)	0.83 (0.30–2.26)	2.12 (1.01–4.42)

Model 1 is the unadjusted model. Model 2 is adjusted with TAC-C0-TWA. Model 3 is adjusted with TAC-C0-TWA and other parameters showing significant differences in baseline characteristics among the four groups according to TAC-IPV and RM/NRM status (^*^age, sex, BMI, induction regimen, ^†^age, sex, induction regimen, ^‡^age, sex, BMI, induction regimen).

BMI, body mass index; DCGL, death-censored graft loss; IPV, intra-patient variability; NRM, non-rapid metabolizer; PRA, panel reactive antibody; Ref., Reference; RM, rapid metabolizer.

## Discussion

The results of the present study revealed that high IPV with RM, which was represented by low CDR, was associated with adverse allograft outcomes, such as acute TCMR, active ABMR, chronic ABMR, and DCGL in immunologically low-risk KTs. This study provides information that patients considered to be RM should pay more attention to controlling TAC-IPV, not only focusing on achieving target TAC-C0.

Since the follow-up interval varies depending on the period after KT, it is necessary to correct the difference in this interval in TAC-IPV calculation (3). Hence, TWCV calculation method, which is a more objective TAC-IPV index, was adopted in the present study (5, 8). Additionally, the frequency of TAC measurements in the high-IPV groups was significantly higher, which was attributed to the fact that the patients with large fluctuations in TAC-C0 undertook more frequent outpatient clinic visits to reach the target TAC-C0 range. Moreover, management of allograft complications might reduce the time interval between outpatient clinic visits, resulting in more frequent TAC measurements. Furthermore, TAC-C0-TWAs were significantly lower in the RM groups. Generally, RMs require a higher drug dose than NRMs to reach the target TAC-C0; however, despite the higher drug doses, the average TAC-C0 can be lower in the RMs than in NRMs (25).

The incidence of BPAR for the overall cohort was higher in the high-IPV groups, and it was the highest for the high-IPV/RM group. When classified according to rejection subtypes, acute TCMR and chronic active ABMR were higher in the high-IPV groups, which is consistent with the findings of the previous studies (7, 26, 27). The incidence of active ABMR was significantly higher in the high IPV/RM group, and this was only observed for the PRA-positive subgroup. This is discussed in the second next paragraph. In addition, a higher tendency for CNI toxicity was observed in the RM group compared to NRM group. Low CDR is a significant risk factor for increased CNI toxicity in another previous study (14), but the difference was not statistically significant in the present study (**Supplemental Table 3**).

The incidence of DCGL was highest in cases of high IPV with RM. RM requires a higher drug dose to reach the same target TAC-C0; therefore, the peak drug blood concentration increases, which may increase drug toxicity. Conversely, owing to the rapid metabolism of TAC in RM, the average TAC-C0 can be low. Hence, a sufficient immunosuppressive effect cannot be obtained (11, 14). High IPV may cause excessive fluctuations in peak-to-trough drug concentrations in RM, which can worsen the allograft prognosis. A low mean TAC-C0 is a well-known risk factor for adverse allograft outcomes (28, 29) and may have an impact on the study results. In present study, TAC-C0-TWAs were significantly lower in the RM groups. Notably, TAC-C0-TWAs were also lower in the RM groups during the first 3 months after KT (7.57 ± 1.34 ng/mL in the low-IPV/NRM group, 8.22 ± 1.71 ng/mL in the high-IPV/NRM group, 6.34 ± 1.24 ng/mL in the low-IPV/RM group, and 6.91 ± 1.44 ng/mL in the high-IPV/RM group; P < 0.001; data not shown). In addition, the proportion of antithymocyte globulin use as an induction regimen was relatively lower in the high-IPV/RM group. It is possible that the low rate of antithymocyte globulin use, a strong induction regimen, in conjunction with the low TAC-C0-TWA in the early post-KT period may have affected the adverse allograft outcome in the high-IPV/RM group. However, high IPV/RM was still identified as an independent risk factor for DCGL even when TAC-C0-TWA and induction regimen were adjusted in the multivariate Cox regression analysis.

This study was performed in patients with low immunological risk without pre-transplant HLA-DSAs. PRA positivity was observed in approximately 30% of the patients, and these patients were considered to have non-donor-specific HLA-antibodies before KT. Further, subgroup analysis was performed for these patients because they had a higher immunological risk than PRA-negative patients. Subgroup analysis based on the pre-transplant PRA status showed that the incidence of active ABMR was considerably higher in the high-IPV/RM group only in the PRA-positive subgroup. A previous study analyzed TAC-IPV according to pre-transplant immunological risk and reported that high IPV is a significant risk factor for ABMR only in the high immunological risk group (PRA ≥ 20% or the presence of DSA); however, they did not analyze the relationship with CDR (30). In RM with low CDR, it might be difficult to reach the target drug blood concentration to ensure adequate immunosuppressive effects. Patients who have non-donor-specific HLA-antibodies before KT may be more vulnerable to inadequate immunosuppression. Hence, excessive fluctuations in TAC blood concentration caused by combined high-IPV may have a significant impact on the increased risk of active ABMR. In addition, the HR for DCGL of high-IPV/RM was increased in the PRA-positive subgroup in all models of the Cox regression analysis.

Whether a low CDR itself is an independent risk factor for adverse allograft outcomes remains controversial. Low CDR has been associated with CNI toxicity, BK viremia, decreased allograft function, and an independent risk factor for DCGL (11, 13, 31). In contrast, some studies have reported that low CDR is not a significant risk factor for adverse allograft outcomes, such as BPAR and DCGL (15, 16). In the present study, a low CDR itself was not observed as an independent risk factor for DCGL when other confounding factors (TAC-C0-TWA, TAC-C0-TWCV, age, and induction regimen, **Supplemental Table 4**) were adjusted (HR 1.66, 95% confidence interval 0.73–3.76). To the best of our knowledge, only one previous study has reported the combined impact of interpatient variability and IPV, and it reported that high IPV with low CDR is a significant risk factor for adverse allograft outcomes. However, they also reported that high IPV itself had no impact on adverse allograft outcomes, which is not consistent with the results of previous TAC-IPV studies. The authors attributed this difference to the small sample size and short-term follow-up period (17). In the present study, we ensured large sample size and used time-interval-corrected TWCV as an objective IPV index. In addition, the observation of long-term allograft outcomes provided adequate and more objective evidence for the combined effects of TAC inter-patient variability and IPV on adverse allograft outcomes.

High IPV in patients with RM showed poor allograft outcomes; therefore, efforts should be made to alleviate them in such patients. It is known that TAC-IPV can be altered by various factors such as drug adherence, absorption, metabolism, formulation, concomitant medications, and comorbidities (32). Therefore, it is difficult to present a clear method for improving TAC-IPV, although drug adherence has been identified as the most important factor to date (33). A recent study reported that active intervention improved TAC-IPV in a randomized controlled trial using an electronic monitoring system with a mobile health application to improve drug adherence (34). No method to change the metabolic state of the RMs has been identified yet. However, a previous ASERTAA study reported that CDRs change depending on the drug formulation, suggesting that a prolonged-release formulation of TAC might be helpful for RMs (35). Further studies that apply different treatment strategies are required to improve the prognosis of these patients.

This study had a few limitations. First, the cause of the high TAC-IPV could not be elucidated as this was a retrospective study. Hence, it was difficult to identify a method to effectively reduce TAC-IPV. In particular, the relationship between TAC-IPV and allograft complications (rejection and infection) is unclear. TAC-IPV may affect the occurrence of allograft complications; however, changes in TAC-IPV may also occur due to changes in the TAC dose prescription due to the primary occurrence of allograft complications. However, because a prospective study is not possible for TAC-IPV, this is a fundamental limitation for all TAC-IPV studies. Second, *de novo* DSA may be underestimated. Contrary to our expectations, there was no difference in the incidence of *de novo* DSA development among the four groups. This may be because we were unable to observed all the *de novo* DSA in our study. *De novo* anti-HLA-DQ antibodies are known to impact chronic active ABMR (26). However, as HLA-DQ typing of kidney donors in our center started 2016 onward, the anti-HLA-DQ antibodies observed in KT recipients before 2016 may not be detected as DSA.

In conclusion, the results of the present study suggest that high IPV with RM shows the worst long-term allograft outcomes. This was observed as a more prominent risk factor in PRA-positive patients with a relatively higher immunological risk. This study suggests the need for individualized treatment strategies by confirming whether the patient has RM in the process of management after KT and stratifying the patient’s risk according to TAC-IPV. Future efforts should be made to improve patient prognosis through strategies to improve TAC-IPV and TAC metabolism state in these patients.

## Data availability statement

The data that support the findings of this study are available on request from the corresponding author. The data are not publicly available due to privacy or ethical restrictions.

## Ethics statement

The studies involving human participants were reviewed and approved by Seoul St. Mary’s Hospital. Written informed consent for participation was not required for this study in accordance with the national legislation and the institutional requirements.

## Author contributions

YP, JS, and BHC wrote the manuscript. CWY, and BHC designed the study. YP and JS performed the experiments. and HL, SHE, EJK, JWM, S-HY, W-MH, S-RY, and JS analyzed the data. All authors contributed to the article and approved the submitted version.

## Funding

This work was supported by a grant from Research Settlement Fund for the new faculty on Konyang University Hospital and the Korean Health Technology R&D Project, Ministry of Health & Welfare, Republic of Korea (HI22C0422).

## Acknowledgments

We thank PYEONGHWA IS Co. for their help in performing this research.

## Conflict of interest

The authors declare that the research was conducted in the absence of any commercial or financial relationships that could be construed as a potential conflict of interest.

## Publisher’s note

All claims expressed in this article are solely those of the authors and do not necessarily represent those of their affiliated organizations, or those of the publisher, the editors and the reviewers. Any product that may be evaluated in this article, or claim that may be made by its manufacturer, is not guaranteed or endorsed by the publisher.
