# Supplementary material for: Combined impact of the inter and intra-patient variability of tacrolimus blood level on allograft outcomes in kidney transplantation
Source: Front Immunol. 2022 Nov 16;13:1037566. doi: 10.3389/fimmu.2022.1037566 (PMC9709474; doi:10.3389/fimmu.2022.1037566)
Supplement: Supplementary file 1 [file DataSheet_1.docx]

# Supplementary Tables

**Table S1.** Baseline characteristics according to TAC-IPV and RM/NRM status in PRA positive subgroup

|  | **Low-IPV /NRM**  **(n = 157)** | **High-IPV /NRM**  **(n = 115)** | **Low-IPV /RM**  **(n = 29)** | **High-IPV /RM**  **(n = 47)** | **P-value** |
| --- | --- | --- | --- | --- | --- |
| **Donor factors** |  |  |  |  |  |
| Age (years) | 44.2 ± 11.9 | 43.9 ± 13.6 | 42.7 ± 13.1 | 44.4 ± 13.0 | 0.956 |
| Male sex (n, %) | 82 (52.2%) | 62 (53.9%) | 16 (55.2%) | 29 (61.7%) | 0.723 |
| BMI (kg/m^2^) | 23.3 ± 3.1 | 23.9 ± 3.5 | 22.6 ± 3.6 | 24.4 ± 3.8 | 0.086 |
| **Transplant information** |  |  |  |  |  |
| Preemptive KT (n, %) | 35 (22.3%) | 18 (15.7%) | 5 (17.2%) | 15 (31.9%) | 0.123 |
| Deceased donor KT (n, %) | 60 (38.2%) | 48 (41.7%) | 9 (31.0%) | 11 (23.4%) | 0.146 |
| ABO-incompatible KT (n, %) | 26 (16.6%) | 24 (20.9%) | 1 (3.4%) | 10 (21.3%) | 0.143 |
| Previous history of KT (n, %) | 4 (2.5%) | 3 (2.6%) | 0 (0.0%) | 3 (6.4%) | 0.388 |
| Cold ischemic time (min) | 182 ± 103 | 184 ± 73 | 254 ± 138 | 244 ± 104 | 0.072 |
| Mismatch number | 3.11 ± 1.60 | 3.38 ± 1.49 | 2.59 ± 1.90 | 3.26 ± 1.66 | 0.192 |
| **Tacrolimus-related information** |  |  |  |  |  |
| Tacrolimus measurement frequency | 15.3 ± 3.0 | 15.9 ± 2.6^‡^ | 14.2 ± 2.8^†^ | 15.9 ± 5.0 | 0.030 |
| TAC-C0-TWA (ng/mL) | 6.94 ± 1.30^†‡§^ | 6.39 ± 1.73^*‡§^ | 5.80 ± 1.18^*†§^ | 5.41 ± 1.44^*†‡^ | <0.001 |
| TWCV (%) | 22.9 ± 4.3^†§^ | 44.2 ± 14.7^*‡^ | 23.2 ± 5.0^†§^ | 41.6 ± 11.5^*‡^ | <0.001 |
| CDR (ng•mg/mL) | 2.16 ± 1.10^‡§^ | 2.10 ± 1.33^‡§^ | 0.86 ± 0.15^*†^ | 0.82 ± 0.16^*†^ | <0.001 |
| **Recipient factors** |  |  |  |  |  |
| Age (years) | 47.9 ± 10.4^§^ | 47.3 ± 11.3 | 46.4 ± 12.9 | 42.5 ± 11.1^*^ | 0.046 |
| Male sex (n, %) | 88 (56.1%) | 47 (40.9%) | 18 (62.1%) | 19 (40.4%) | 0.023 |
| BMI (kg/m^2^) | 22.6 ± 3.3 | 22.2 ± 3.0 | 22.4 ± 3.7 | 21.8 ± 3.7 | 0.428 |
| Cause of ESKD |  |  |  |  |  |
| DM (n, %) | 27 (17.2%) | 25 (21.7%) | 6 (20.7%) | 6 (12.8%) | 0.552 |
| HTN (n, %) | 20 (12.7%) | 14 (12.2%) | 3 (10.3%) | 2 (4.3%) | 0.426 |
| CGN (n, %) | 53 (33.8%) | 39 (33.9%) | 16 (55.2%) | 22 (46.8%) | 0.066 |
| Others (n, %) | 26 (16.6%) | 12 (10.4%) | 2 (6.9%) | 4 (8.5%) | 0.233 |
| Unknown (n, %) | 31 (19.7%) | 25 (21.7%) | 2 (6.9%) | 13 (27.7%) | 0.176 |
| Dialysis information |  |  |  |  |  |
| Hemodialysis (n, %) | 100 (63.7%) | 83 (72.2%) | 19 (65.5%) | 26 (55.3%) | 0.199 |
| Peritoneal dialysis (n, %) | 22 (14.0%) | 14 (12.2%) | 5 (17.2%) | 6 (12.8%) | 0.902 |
| Dialysis vintage (months) | 60.2 ± 63.3^‡^ | 41.1 ± 43.6 | 27.5 ± 39.3^*^ | 37.4 ± 44.0 | 0.013 |
| **Induction regimen** |  |  |  |  |  |
| Antithymocyte globulin (n, %) | 59 (37.6%)^†^ | 23 (20.0%)^*^ | 7 (24.1%) | 11 (23.4%) | 0.011 |
| Basiliximab (n, %) | 98 (62.4%)^†^ | 92 (80.0%)^*^ | 22 (75.9%) | 36 (76.6%) | 0.011 |

Continuous variables are shown as mean ± standard deviation and categorical variables are shown as proportions. ^*^P <0.0083 versus low-IPV/NRM group, ^†^P <0.0083 versus high-IPV/NRM group, ^‡^P <0.0083 versus low-IPV/RM group, ^§^P <0.0083 versus high-IPV/RM group
BMI, body mass index; CDR, concentration-to-dose ratio; CGN, clinical glomerulonephritis; DM, diabetes mellitus; ESKD, end-stage kidney disease; HTN, hypertension; IPV, intra-patient variability; KT, kidney transplantation; NRM, non-rapid metabolizer; PRA, panel reactive antibody; RM, rapid metabolizer; TAC, tacrolimus; TAC-C0, tacrolimus trough level; TWA, time-weighted average; TWCV, time-weighted coefficient variation

**Table S2.** Baseline characteristics according to TAC-IPV and RM/NRM status in PRA negative subgroup

|  | **Low-IPV /NRM**  **(n = 295)** | **High-IPV /NRM**  **(n = 260)** | **Low-IPV /RM**  **(n = 77)** | **High-IPV /RM**  **(n = 100)** | **P-value** |
| --- | --- | --- | --- | --- | --- |
| **Donor factors** |  |  |  |  |  |
| Age (years) | 45.0 ± 12.7 | 45.5 ± 12.6 | 44.8 ± 12.3 | 44.6 ± 14.2 | 0.954 |
| Male sex (n, %) | 143 (48.5%) | 128 (49.2%) | 41 (53.2%) | 50 (50.0%) | 0.903 |
| BMI (kg/m^2^) | 23.7 ± 3.6 | 23.5 ± 3.3 | 24.1 ± 3.7 | 23.6 ± 3.7 | 0.696 |
| **Transplant information** |  |  |  |  |  |
| Preemptive KT (n, %) | 63 (21.4%) | 64 (24.6%) | 14 (18.2%) | 20 (20.0%) | 0.573 |
| Deceased donor KT (n, %) | 94 (31.9%) | 92 (35.4%) | 28 (36.4%) | 31 (31.0%) | 0.721 |
| ABO-incompatible KT (n, %) | 43 (14.6%) | 32 (12.3%) | 12 (15.6%) | 16 (16.0%) | 0.757 |
| Previous history of KT (n, %) | 7 (2.4%) | 2 (0.8%) | 1 (1.3%) | 3 (3.0%) | 0.381 |
| Cold ischemic time (min) | 191 ± 90 | 190 ± 75 | 189 ± 63 | 201 ± 74 | 0.930 |
| Mismatch number | 3.63 ± 1.49 | 3.46 ± 1.61 | 3.56 ± 1.43 | 3.58 ± 1.42 | 0.610 |
| **Tacrolimus-related information** |  |  |  |  |  |
| Tacrolimus measurement frequency | 15.0 ± 2.8^†§^ | 15.6 ± 3.2^*‡^ | 15.2 ± 2.2^†§^ | 16.0 ± 2.9^*‡^ | <0.001 |
| TAC-C0-TWA (ng/mL) | 6.93 ± 1.18^†‡§^ | 6.54 ± 1.70^*‡§^ | 6.00 ± 1.72^*†§^ | 5.16 ± 1.41^*†‡^ | <0.001 |
| TWCV (%) | 22.6 ± 4.6^†§^ | 43.3 ± 12.7^*‡^ | 23.4 ± 4.3^†§^ | 41.7 ± 10.6^*‡^ | <0.001 |
| CDR (ng•mg/mL) | 2.17 ± 1.17^‡§^ | 2.14 ± 1.01^‡§^ | 0.81 ± 0.16^*†^ | 0.79 ± 0.17^*†^ | <0.001 |
| **Recipient factors** |  |  |  |  |  |
| Age (years) | 49.0 ± 11.5^§^ | 46.8 ± 12.0^§^ | 45.5 ± 10.1 | 42.5 ± 11.9^*†^ | <0.001 |
| Male sex (n, %) | 214 (72.5%)^†^ | 159 (61.2%)^*^ | 58 (75.3%) | 70 (70.0%) | 0.015 |
| BMI (kg/m^2^) | 23.6 ± 3.7 | 23.2 ± 3.6 | 24.3 ± 3.5 | 23.2 ± 3.4 | 0.043 |
| Cause of ESKD |  |  |  |  |  |
| DM (n, %) | 69 (23.4%) | 51 (19.6%) | 20 (26.0%) | 18 (18.0%) | 0.424 |
| HTN (n, %) | 41 (13.9%) | 41 (15.8%) | 16 (20.8%) | 17 (17.0%) | 0.503 |
| CGN (n, %) | 101 (34.2%) | 94 (36.2%) | 20 (26.0%) | 30 (30.0%) | 0.334 |
| Others (n, %) | 33 (11.2%) | 29 (11.2%) | 7 (9.1%) | 14 (14.0%) | 0.775 |
| Unknown (n, %) | 51 (17.3%) | 45 (17.3%) | 14 (18.2%) | 21 (21.0%) | 0.851 |
| Dialysis information |  |  |  |  |  |
| Hemodialysis (n, %) | 175 (59.3%) | 150 (57.7%) | 49 (63.6%) | 54 (54.0%) | 0.610 |
| Peritoneal dialysis (n, %) | 57 (19.3%) | 46 (17.7%) | 14 (18.2%) | 26 (26.0%) | 0.345 |
| Dialysis vintage (months) | 38.3 ± 49.8 | 45.0 ± 52.2 | 40.2 ± 52.3 | 36.9 ± 55.2 | 0.584 |
| **Induction regimen** |  |  |  |  |  |
| Antithymocyte globulin (n, %) | 32 (10.9%) | 34 (14.2%) | 9 (11.7%) | 5 (5.0%) | 0.101 |
| Basiliximab (n, %) | 263 (89.2%) | 226 (86.9%)^§^ | 69 (89.6%) | 97 (97.0%)^†^ | 0.101 |

Continuous variables are shown as mean ± standard deviation and categorical variables are shown as proportions. ^*^P <0.0083 versus low-IPV/NRM group, ^†^P <0.0083 versus high-IPV/NRM group, ^‡^P <0.0083 versus low-IPV/RM group, ^§^P <0.0083 versus high-IPV/RM group
BMI, body mass index; CDR, concentration-to-dose ratio; CGN, clinical glomerulonephritis; DM, diabetes mellitus; ESKD, end-stage kidney disease; HTN, hypertension; IPV, intra-patient variability; KT, kidney transplantation; NRM, non-rapid metabolizer; PRA, panel reactive antibody; RM, rapid metabolizer; TAC, tacrolimus; TAC-C0, tacrolimus trough level; TWA, time-weighted average; TWCV, time-weighted coefficient variation

**Table S3.** Primary and secondary outcomes according to RM/NRM status

|  | **RM**  **(n = 253)** | **NRM**  **(n = 827)** | **P-value** |
| --- | --- | --- | --- |
| **DCGL** | 34 (13.4%) | 63 (7.6%) | 0.005 |
| **Overall graft loss** | 45 (17.8%) | 94 (11.4%) | 0.008 |
| **Overall BPAR** | 66 (26.1%) | 146 (17.7%) | 0.003 |
| Acute TCMR | 53 (21.0%) | 119 (14.4%) | 0.013 |
| Active ABMR | 19 (7.5%) | 29 (3.5%) | 0.007 |
| Chronic active TCMR | 1 (0.4%) | 9 (1.1%) | 0.314 |
| Chronic active ABMR | 7 (2.8%) | 21 (2.5%) | 0.842 |
| **De novo DSA positive** | 35 (13.8%) | 95 (11.5%) | 0.316 |
| **CNI toxicity** | 49 (19.4%) | 126 (15.4%) | 0.119 |
| **BK virus nephropathy** | 9 (3.6%) | 29 (3.5%) | 0.970 |

Categorical variables are shown as proportions.
ABMR, antibody-mediated rejection; BPAR, biopsy proven acute rejection; CNI, calcineurin inhibitor; DCGL, death-censored graft loss; DSA, donor-specific antibody; NRM, non-rapid metabolizer; RM, rapid metabolizer; TCMR, T-cell mediated rejection

**Table S4.** Baseline characteristics according to RM/NRM status

|  | **RM**  **(n = 253)** | | **NRM**  **(n = 827)** | | **P-value** |
| --- | --- | --- | --- | --- | --- |
| **Donor factors** |  |  |  |  |  |
| Age (years) | 44.4 ± 13.2 | | 44.8 ± 12.6 | | 0.660 |
| Male sex (n, %) | 136 (53.8%) | | 413 (49.9%) | | 0.320 |
| BMI (kg/m^2^) | 23.8 ± 3.7 | | 23.6 ± 3.4 | | 0.472 |
| **Transplant information** |  |  |  |  |  |
| Preemptive KT (n, %) | 54 (21.3%) | | 180 (21.8%) | | 0.887 |
| Deceased donor KT (n, %) | 79 (31.2%) | | 294 (35.6%) | | 0.206 |
| ABO-incompatible KT (n, %) | 39 (15.4%) | | 125 (15.1%) | | 0.765 |
| Previous history of KT (n, %) | 7 (2.8%) | | 16 (1.9%) | | 0.422 |
| PRA positive (n, %) | 76 (30.0%) | | 272 (32.9%) | | 0.394 |
| Mismatch number | 3.40 ± 1.55 | | 3.44 ± 1.56 | | 0.699 |
| **Tacrolimus-related information** |  |  |  |  |  |
| Tacrolimus measurement frequency | 15.5 ± 3.2 | | 15.4 ± 3.0 | | 0.394 |
| TAC-C0-TWA (ng/mL) | 5.53 ± 1.53 | | 6.74 ± 1.48 | | <0.001 |
| TWCV (%) | 34.0 ± 12.6 | | 32.2 ± 14.1 | | 0.068 |
| CDR (ng•mg/mL) | 0.81 ± 0.16 | | 2.15 ± 1.13 | | <0.001 |
| **Recipient factors** |  |  |  |  |  |
| Age (years) | 43.8 ± 11.4 | | 47.9 ± 11.4 | | <0.001 |
| Male sex (n, %) | 165 (65.2%) | | 508 (61.4%) | | 0.276 |
| BMI (kg/m^2^) | 23.2 ± 3.6 | | 23.1 ± 3.5 | | 0.702 |
| Cause of ESKD |  |  |  |  |  |
| DM (n, %) | 50 (19.8%) | | 172 (20.8%) | | 0.721 |
| HTN (n, %) | 38 (15.0%) | | 116 (14.0%) | | 0.111 |
| CGN (n, %) | 88 (34.8%) | | 287 (34.7%) | | 0.096 |
| Others (n, %) | 27 (10.7%) | | 100 (12.1%) | | 0.117 |
| Unknown (n, %) | 50 (19.8%) | | 152 (18.4%) | | 0.636 |
| Dialysis information |  |  |  |  |  |
| Hemodialysis (n, %) | 148 (58.5%) | | 508 (61.4%) | | 0.404 |
| Peritoneal dialysis (n, %) | 51 (20.2%) | | 139 (16.8%) | | 0.221 |
| Dialysis vintage (months) | 37.1 ± 50.8 | | 45.0 ± 53.1 | | 0.090 |
| **Induction regimen** |  |  |  |  |  |
| Antithymocyte globulin (n, %) | 32 (12.6%) | | 151 (18.3%) | | 0.037 |
| Basiliximab (n, %) | 226 (89.3%) | | 679 (82.1%) | | 0.006 |

Continuous variables are shown as mean ± standard deviation and categorical variables are shown as proportions.
BMI, body mass index; CDR, concentration-to-dose ratio; CGN, clinical glomerulonephritis; DM, diabetes mellitus; ESKD, end-stage kidney disease; HTN, hypertension; KT, kidney transplantation; NRM, non-rapid metabolizer; PRA, panel reactive antibody; RM, rapid metabolizer; TAC, tacrolimus; TAC-C0, tacrolimus trough level; TWA, time-weighted average; TWCV, time-weighted coefficient variation
